# Impact of Sugar Type Addition and Fermentation Temperature on Pomegranate Alcoholic Beverage Production and Characteristics

**DOI:** 10.3390/antiox10060889

**Published:** 2021-06-01

**Authors:** Evangelos Kokkinomagoulos, Anastasios Nikolaou, Yiannis Kourkoutas, Costas G. Biliaderis, Panagiotis Kandylis

**Affiliations:** 1 Department of Food Science and Technology, School of Agriculture, Aristotle University of Thessaloniki, P.O. Box 235, 54124 Thessaloniki, Greece; ekokkinom@gmail.com (E.K.); biliader@agro.auth.gr (C.G.B.); 2 Laboratory of Applied Microbiology & Biotechnology, Department of Molecular Biology & Genetics, Democritus University of Thrace, 68100 Alexandroupolis, Greece; anikol@mbg.duth.gr (A.N.); ikourkou@mbg.duth.gr (Y.K.)

**Keywords:** pomegranate, alcoholic fermentation, phenolics, antioxidant activity, aromatic compounds, bioactive compounds, flavonoids, anthocyanin, GC/MS, *Saccharomyces*

## Abstract

The present study focuses on the production of pomegranate alcoholic beverage (PAB) from juice of the Wonderful variety. The effect of fermentation temperature (15 and 25 °C) and type of sugar added (adjustment to 20 °Brix) on the physicochemical characteristics, bioactive compounds, and volatile composition were studied. Sucrose, concentrated pomegranate juice, concentrated grape juice, and honey were used to increase the initial sugar content. The produced PABs contained ethanol in concentrations ranging from 7.9 to 10.0% *v*/*v* and glycerol from 4.8 to 6.1 g L^−1^. A decrease in total phenolics content, free radical-scavenging activity, and total monomeric anthocyanin content was observed following fermentation. Total flavonoids content appeared to increase after fermentation only in the cases of concentrated pomegranate and grape juice addition. In general, 22 volatile compounds were identified in PABs (13 esters, 2 fatty acids, and 7 alcohols). Major compounds detected were 3-methyl-1-butanol, 2-methyl-1-butanol, 2-phenylethanol, and ethyl acetate. These findings demonstrate the production prospect of PABs with increased ethanol content, while elaborating on the importance of fermentation temperature and the differences between the selected types of added sugars on end-product composition.

## 1. Introduction

Pomegranate (*Punica granatum* L.) is a well-known fruit that has been cultivated and consumed since ancient times. It has been extensively studied for its numerous beneficial effects that its consumption may have on humans, such as anticarcinogenic, antioxidant, and anti-inflammatory, while its applications in food products have also been investigated [1,2,3]. Furthermore, its extracts have been proven capable of possessing antimicrobial activity against several food borne pathogens [4,5]. Additionally, due to the increasing awareness of consumers concerning the possible beneficial health effects of the fruit, pomegranate has emerged as a valuable source of nutrients and its popularity has been rising worldwide. However, pomegranate is currently being consumed not only as fresh fruit and juice, but also as pomegranate alcoholic beverage (PAB), known as “pomegranate wine”, a product of alcoholic fermentation of pomegranate juice. This is in accordance with the growing trend for the development of fermented and not fermented products based on fruit juices [6,7].

During processing of fruit products, procedures, such as pasteurization, can have a negative impact on the aromatic (flavor) intensity of fresh fruit, which, in the case of pomegranate, is considered to be relatively low. Nevertheless, these processes may be of high significance as far as consumer acceptance is concerned. The low aromatic intensity of pomegranate has rendered the study of its volatile aromatic compounds difficult, with various authors presenting different results on the volatile composition of pomegranate and its products. Regarding fresh pomegranate fruit, literature data suggest that hexanal, limonene, 2-hexanal, and α-terpineol are the major aromatic compounds [8], while regarding pomegranate juice, studies suggest that 3-methyl butanal, ethyl butyrate, isopentyl acetate, hexanol, diethyl allyl malonate, and α-ionone are the respective major volatiles [9]. Studies about PABs and their aromatic profile are limited, with current reports indicating ethyl esters such as ethyl hexanoate, ethyl octanoate and ethyl decanoate, ethanol, 3-methyl-1-butanol, phenylethyl alcohol, and 3-methyl-1-butanol acetate, as the major aromatic compounds [10,11].

Fermentation is considered as a process that can alter the sensorial and nutritional properties of a material, and therefore different fermentation conditions and vinification methods may result in the production of distinctive products, even though originating from the same raw plant matrix [12]. The fact that fermentation can lead to completely new products has been proven through the study of bioactive compounds of pomegranate, and especially their chemical composition and characteristics before and after fermentation, possibly governed by a number of chemical and biochemical reactions, such as oxidation, polymerization, hydrolysis, condensation, and others [13]. In addition, it is well-known that yeast fermentation of anthocyanin-rich plant materials results in various transformations of anthocyanins, both physical and chemical, that greatly contribute to the organoleptic properties of the final product and to color stabilization [14]. Aside from the fermentation temperature and microorganism selection, which appear to be the most important factors for fermentation, the addition of sugars can also contribute to the production of a distinctive product in terms of quality attributes. In alcoholic beverage fermentation, the increase of total soluble solids is a common practice aiming at the uniformity of raw materials as fermentable substrates. However, it can also be seen as a process that can introduce additional organoleptic characteristics into a fermentation end-product. As far as PABs are concerned, relevant literature on the fermentation of pomegranate juice fortified with sugars is limited to sucrose [11]. Whereas, typically, the ethanol content of PABs using natural pomegranate juice ranges between 5.6–7.0 *v*/*v*, an increased ethanol content, up to 10%, is rather desired [10,15].

Driven by the recently set legislative framework regarding PABs in Greece (OGG No. 2161/12.06.2018), the present study focused on the production of PABs with increased ethanol content (up to 10% *v*/*v*). The impact of added sugars and fermentation temperature on total phenolics content (TPC), total flavonoids content (TFC), DPPH free radical-scavenging activity (FRSA), physicochemical characteristics, such as pH and acidity (total and volatile), reducing sugars, ethanol, and glycerol content, as well as on the aromatic profiles of PABs was explored. Fermentations were conducted at 15 °C and 25 °C, while the sugar content increase was achieved through the addition of sucrose (S), concentrated pomegranate juice (CPJ), concentrated grape juice (CGJ), and honey (H). To the best of our knowledge, this study is the first to focus on the effects of juice fortification with different types of sugars, as well as, fermentation temperature on the characteristics of PABs.

## 2. Materials and Methods

### 2.1. Pomegranate Juice, Yeast Strain, and Types of Added Sugars

Commercial, 100% natural, pasteurized pomegranate juice from non-concentrated juice (En Karpo, Thessaloniki, Greece) was used in the present study. Commercial *Saccharomyces bayanus* yeast strain was also employed (Craft Series SN9-Red Wine, Mangrove Jack’s, Albany, Auckland, New Zealand). Sucrose (100% granulated table sugar, Belbake, Aachen, Germany), concentrated grape juice (Roditis variety, ~35 °Baumé), and honey (flower honey, Melinda Ltd., Thessaloniki, Greece) were used. Concentrated pomegranate juice (70 °Brix) was obtained through concentration of the pomegranate juice in a vacuum rotary evaporator (Rotavapor R-3000, BUCHI, Flawil, Switzerland) at 30 °C.

### 2.2. Fermentation and Sugar Adjustment of Pomegranate Juice

Increase of total soluble solids of the base pomegranate juice from 14.5 °Brix to 20 °Brix was performed through the addition of four different fermentable materials in the following amounts (per liter of juice): S: 55 g, CPJ: 100 g, CGJ: 100 g, and H: 70 g. Yeast was introduced to aliquots of 200 mL of pomegranate juice (after the addition of sugars) following the manufacturer’s instructions, resulting in 2.4 × 10^5^ CFU mL^−1^. Fermentations took place at 15 °C for 21 days and at 25 °C for 14 days in sterile glass containers (with airlocks containing ethanol 70% *v*/*v*). Fermentations were conducted in triplicate and their progress was monitored by measuring weight loss.

### 2.3. Determination of Reducing Sugars, Ethanol, and Glycerol Content

Reducing sugars (RS) were determined using the DNS (3,5-dinitrosalicylic acid) method [16] with some modifications, as described by Kokkinomagoulos et al. [10]. Ethanol and glycerol contents were determined by means of HPLC, following the method described by Nikolaou et al. [17].

### 2.4. pH, Volatile Acidity, and Total Acidity

pH was measured using a portable, electronic pH-meter (SensoDirect pH 110, AQUALYTIC, Dortmund, Germany).

Total (TA) and volatile acidity (VA) were determined using the OIV-MA-AS313-01 and OIV-MA-AS313-02 method, respectively [18].

### 2.5. Total Flavonoids Content, Total Phenolics Content, Free Radical-Scavenging Activity and Total Monomeric Anthocyanin Content

Total flavonoids content (TFC), total phenolics content (TPC), free radical-scavenging activity (FRSA), and total monomeric anthocyanin content (TMAC) were determined as described in a previous study [10]. More specifically, TFC was determined by the flavonoid-aluminum chloride (AlCl_3_) complexation method and the results were expressed as mg of quercetin equivalents (QE) per liter. TPC was determined by the Folin–Ciocalteau method and the results were expressed as mg gallic acid equivalents (GAE) per liter. FRSA was determined using the free radical DPPH· (2,2 diphenyl-1-picrylhydrazyl) method and the results were expressed as mM Trolox equivalents (TRE). TMAC was determined using the pH differential method and the results were expressed as mg cyanidin-3-glucoside equivalents (Cy3GE) per liter.

### 2.6. HS-SPME GC/MS Analysis

PAB and juice samples were subjected to headspace solid-phase microextraction (HS-SPME) GC/MS analysis using a GC/MS (6890N GC, 5973 NetworkedMS MSD, Agilent Technologies, Santa Clara, CA, USA) equipped with a HP-5MS column (30 m, 0.25 mm i.d., 0.25 μm film thickness, Agilent Technologies, Santa Clara, CA, USA) as recently described [19].

### 2.7. Statistical Analysis

The results were analyzed statistically by ANOVA. Significance level was set at *p* < 0.05. Tukey’s honest significant difference (HSD) test was used to determine significant differences among results (Statistica version 12.0, StatSoft Inc., Tulsa, OK, USA). The principal component analysis (PCA) algorithm was computed using XLSTAT 2015.1.

## 3. Results and Discussion

### 3.1. Physicochemical Characteristics

The fortification of pomegranate juice with several sugar types led to similar initial reducing sugar content (196–200 g L^−1^). The effect of both fermentation temperature and sugar type on reducing sugars was significant (Table 1). Moreover, it appeared that the addition of CGJ also led to higher remaining reducing sugars after fermentation in comparison with the rest of the fortification treatments at both temperatures, indicating that sugars were not fully utilized. As a result, the fortified raw material with CGJ resulted in the lowest ethanol content (7.9–8.9% *v*/*v*) in the fermented product as the respective residual reducing sugars were the highest (7.5–8.4 g D-glucose L^−1^). However, this did not apply for the medium fortified with H, where the final ethanol yield was high (9.8–10.0% *v*/*v*). Statistically, no significant effect was found in the case of final ethanol content. According to Kokkinomagoulos et al. [10], the final ethanol content of PABs with no added sugars ranged between 6.8–7.0% *v*/*v* (using the same yeast and fermentation temperature), values evidently lower in comparison with those presented in Table 1.

Similar levels for glycerol (a non-volatile alcohol that solely impacts taste and texture, elevating the levels of sweetness and fullness of the fermented beverage [20]) content (4.8–6.1 g L^−1^) were noted, and only in the case of CGJ addition the effect of temperature was significant (*p* < 0.05). Comparing the aforementioned results with studies on PABs without any addition of sugars [10], it appears that fortification of the initial substrates with fermentable sugars from different sources did not lead to an increase in glycerol yield proportionate to that of ethanol. Moreover, as in the case of ethanol, glycerol content was not affected by fermentation temperature and sugar type.

The pH is a critical parameter for all types of fermentations, as it has been shown to affect the growth of yeasts as well as the characteristics of the final product, such as color and taste; a pH range of 2.8–4.0 is considered to be ideal [15,21,22]. The pH of the juice employed in the present work (pH 3.12) was slightly lower in comparison to previous studies (pH 3.20–3.50). However, this parameter for PAB can be greatly affected by variety and fruit maturity (e.g., juice from the Wonderful variety possesses a pH of around 2.98). The results presented in Table 1 indicate that the increase in fermentation temperature has led to higher pH values in the end-products. The addition of CGJ led to higher pH values (3.17–3.29) in comparison with the initial pomegranate juice (3.12 ± 0.01), while in all cases, the effects of sugar type addition and temperature were significant.

All substrate fortifications resulted in similar values of volatile acidity after fermentation (0.8–0.9 g acetic acid L^−1^), which were significantly higher than the value of the initial juice (0.1 g acetic acid L^−1^), as volatile acids are being produced during the alcoholic fermentations. The addition of H led to even higher volatile acidity values (1.2 g acetic acid L^−1^) following fermentation at 15 °C.

Finally, concerning total acidity, the addition of CPJ brought about significant effects. The increased levels in total acidity might be attributed to the concentrated nature of the fermentable sugars and organic acids present in the materials used for fortification of the UPJ.

### 3.2. Antioxidant Activity and Phenolic Compounds

Current literature on pomegranate juice suggests that it may contain varying amounts of flavonoids ranging from 45 to 636 mg QE L^−1^ [23,24]. Indeed, the pomegranate juice used in this study appears to confirm those findings (530.1 ± 7.3 mg QE L^−1^). Moreover, the analytical data indicate that the addition of CPJ and CGJ has led to an increase in TFC, a fact that can be attributed to the high content of these matrices in flavonoids, as the addition of S and H did not lead to increased values in comparison with the initial raw material, the unfermented juice. The highest values (Table 2) were obtained with the addition of CGJ (25 °C, 886.3 ± 21.9) and the lowest with the addition of S (15 °C, 272.6 ± 20.4). However, it appears that flavonoids generally tend to decrease during and after fermentation, as other studies suggest, both in pomegranate fermented beverages [10,15] and in white wine fermented without skin contact [25]. The observed effects of the addition of fermentable sugars followed the same pattern at both temperatures, with higher values noted at 25 °C. These trends can probably be explained by the longer fermentation time required at 15 °C. All effects were significant in all cases of fortification and fermentation temperature.

Phenolic compounds are considered to play an important role in the sensorial perception of a food product, including juices and fermented alcoholic beverages, mostly due to their ability to alter aroma, color, and flavor [26]. None of the substrate fortification regimes led to an increase in TPC following fermentation in comparison with the initial juice. However, compared to PABs produced using unfortified pomegranate juice (807–834 mg GAE L^−1^) [10], the results of the present study were significantly higher, especially in the case of CPJ and CGJ. These results were due to the high TPC of the added CPJ and CGJ. Indeed, the addition of S did not alter the initial TPC of the juice, while a slight increase was noted for the respective fortified substrate (2700 mg GAE L^−1^) in the case of H, due to its TPC of up to 1500 mg GAE kg^−1^ or even higher [27,28]. Finally, the addition of concentrated juices, either pomegranate or grape, resulted in the highest initial TPC of >3000 mg GAE L^−1^. This explains the significant effect of sugar type on the final TPC of PABs (Table 2). The effect of temperature was also significant, with differences of about 400–500 mg GAE L^−1^ being noted in the final values between the two temperature regimes (i.e., the lower contents of phenolics were noted at 25 °C). Apparently, this trend for the TPC was the opposite to that found for the flavonoids (Table 2). Consequently, it can be deduced that pomegranate flavonoids are less sensitive when fermentation is carried out at 25 °C and are degraded at a slower rate in comparison with other phenolics. For the latter, the pronounced decreases can possibly be the result of hydrolysis, oxidation, polymerization, and condensation reactions the polyphenols undergo during the fermentation stage, as well as the adsorption of phenolics to the yeast cells [26,29]. Sugar type also significantly affected the TPC; i.e., addition of S and H resulted in lower values of TPC, compared to fortifications with the concentrated juices (CGJ and CPJ). In a recent study, a decrease in TPC has been also reported for PABs [10].

Determination of free radical-scavenging activity (FRSA) through DPPH· inhibition represents the most common method for determination of antioxidant activity in plant materials, including pomegranate [30]. It is also a convenient method for evaluation of the antioxidant activity of fermented products [31,32,33]. Due to the fact that many studies on pomegranate and other fruit juices have utilized this method, for such measurements [15,34,35,36] or even in combination with other methods [37,38], it was adopted in the present work in order to facilitate comparisons with relevant literature information. The FRSA appeared to be higher at the end of fermentations at 25 °C in comparison with 15 °C, with all fortification treatments leading to significantly different results. The highest values were observed in the cases of CPJ addition (19.3 and 19.8 mM TRE), followed by the addition of CGJ (14.2 and 15.1 mM TRE), H (14.1 and 14.6 mM TRE), and finally S (13.8 and 14.2 mM TRE). Akalin et al. [39] studying fermented pomegranate beverages with no sugar addition, reported lower antioxidant activities (9.5–9.9 mM TRE). Lower FRSA values in PABs have been previously reported [10] and can be attributed to oxidation reactions taking place between phenolic compounds and other molecules present in the composite beverage matrix, bringing about reduced antioxidant potencies [15]. As in the case of TPC and TFC, the results of FRSA are also related to the initial FRSA of the fortified juices. The addition of CPJ resulted in the highest initial FRSA followed by CGJ, while S and H did not affect FRSA, due to their low content compared to pomegranate juice.

The TMAC of the final products seemed to be higher after fermentation at 15 °C, with fortification treatments and temperatures leading to significantly different results. The addition of CPJ resulted in significantly higher TMAC both before (200 mg Cy3GE L^−1^) and after fermentation (131.5–144.0 mg Cy3GE L^−1^). It can be concluded that the FRSA of fermented PABs is primarily due to non-anthocyanin flavonoid compounds, as higher FRSA was noted in the cases where TFC was higher and the TMAC lower. This observation does not comply with the findings from other studies (e.g., Ghiselli et al. [40] reported that the fraction of anthocyanins exhibited higher antioxidant activity in comparison with other phenolic fractions). Polymerization of monomeric anthocyanins could be responsible for the decrease in TMAC following fermentation, since polymerized anthocyanins do not undergo a color change with pH alteration when present in solutions, thus absorbing at both wavelengths used in the pH-differential method employed for their determination. Additionally, this decrease could also be attributed to degradation reactions (such as oxidation and hydrolysis) and interactions between anthocyanins and other phenolics [41]. Previous studies on the antioxidant activity and phenolic compounds of PABs have pointed to similar or even lower values for these quality characteristics [10,11,15,39,42], implying that the addition of different types of fermentable sugar sources may improve the final products in terms of increased phenolics content and antioxidant activity.

### 3.3. Volatile Composition

It is well-known that commercially pasteurized juices possess relatively weaker aromatic profiles compared to their fresh counterparts, underlying mostly the negative effects thermal treatments (e.g., pasteurization) have on volatile constitutes. Therefore, in the present study, the use of commercial pasteurized juices led to low initial volatile content (Table S1). Similar results were observed in our previous study [10] and in other studies using pasteurized juices [43,44]. However, this is ideal for the aims of the present study, since the volatiles detected in PABs are correlated with yeast metabolism, fermentation temperature, and sugar type added.

In PABs, a sum of 22 different volatile compounds were identified, out of which 13 were esters, 2 fatty acids, and 7 alcohols (Table 3). Among these, the most dominant group was alcohols, followed by esters and fatty acids. It appears that, for the majority of samples, fermentations at 15 °C resulted in increased production of aromatic constituents, compared with fermentations carried out at 25 °C, with the exception of CGJ addition, where the opposite was noted. In general, the volatile profiles of PABs were quite different from those of juices, as has been also reported in similar studies [45].

Although there is no literature information on the volatile composition of PABs with increased ethanol content, the majority of identified volatiles are confirmed by other relevant studies on pomegranate and its products [10,44,45,46]. However, there are many factors that can affect the presence and concentration of volatile compounds such as genotype and fruit chemical composition, harvesting, and fermentation techniques, environmental conditions, etc. [47].

#### 3.3.1. Esters

Esters comprise a group of volatile constituents that are present in fruits and vegetables and to which the fruity notes in these products can be attributed. In this group of compounds, ethyl acetate is one of the most dominant aromatic constituents, contributing to the sweet-fruity odor. Its presence has been previously reported in pomegranate juice [43], whereas the intense berry and fruity odor of pomegranate has also been attributed to esters, mainly ethyl acetate and octyl acetate [45]. In the present study, ethyl acetate was found to be the most abundant ester, being detected in all samples with higher concentrations in the products obtained by fermentation at lower temperatures. In addition, it appeared that only the effect of fermentation temperature was significant concerning the concentration of 2-phenylethyl acetate, ranging between 0.35 and 1.35 mg L^−1^. This compound is thought to be a key volatile of pomegranate arils, and is linked with flowery, fruity, and cooked apple aroma notes [48]. Moreover, 3-methylbutyl acetate and 2-methylbutyl acetate were detected in all samples, with the first being present at higher concentrations. These compounds have been previously found in pomegranate juice and are thought to intensify the fruity aroma [43,49]. In general, PABs fermented with added S and CPJ presented the highest concentrations of esters at 15 and 25 °C, respectively. Statistical analysis did reveal a significant effect of sugar type on the total concentrations of esters in PABs.

#### 3.3.2. Fatty Acids

Fatty acids are also crucial for the development of flavor and taste in beverages. Firstly, they can contribute to sourness, while possessing their own characteristic aroma [50]. They are considered as having an impact on the complexity of aroma even at concentrations below their odor detection thresholds [51,52]. Octanoic and decanoic acids that have been identified in the PAB samples have been reported as the most abundant acids in wines and cider [17,53,54,55]; decanoic acid was identified in lower concentrations and only in certain samples. In all cases, no significant differences were observed (*p* > 0.05). The group of fatty acids (organic acids), especially octanoic and decanoic acid, is characteristic in PABs, while it is not detected in the initial juices [45]. Therefore, they are derived from yeast fermentation.

#### 3.3.3. Alcohols

Alcohols are important aromatic constituents of fermented beverages as they substantially contribute to the odor intensity, despite the fact that they generally tend to impart unpleasant odors [51]. Fermentation temperature and sugar type had no significant (*p* > 0.05) effect in the present study. The most dominant volatile alcohol (recognized as aroma-related compound) appeared to be 3-methyl-1-butanol; this compound, being responsible for a whiskey malt, burnt aroma, has been isolated from pomegranate stem peels [56]. A higher alcohol, 2-phenylethanol (fusel alcohol) with a pleasant, old rose odor was also identified in the PABs, with concentrations ranging from 2.00 to 9.85 mg L^−1^ [51,57]. Previous studies have reported that 2-phenylethanol accounts for approximately 40% of the total bound aromatic compounds of pomegranate [58], and it has been also related with the aroma notes of other fermented beverages, such as red wine vinegars [59] and Muscat of Bornova wines [60]. Among other alcohols, 2,3-butanediol, (Z)-3-hexen-1-ol and 1-hexanol, also contributing to the aroma of pomegranate products, were generally detected in low concentrations, if not at all, in some cases. 2,3-Butanediol has been previously identified in commercial pomegranate juice and has been linked with fruit and onion aroma [44]. Furthermore, (Z)-3-hexen-1-ol (grass aroma) has been identified as a major aromatic compound in pomegranate seeds [61], and in pomegranate juice [46,62]. Finally, in other studies, the presence of 1-hexanol in pasteurized pomegranate products has been reported [43,44].

#### 3.3.4. Chemometrics

The principal component analysis algorithm was applied to all HS-SPME GC/MS data, revealing only significant differences between initial juices and PABs (Figure S1). In general, as it was also evident from the GC/MS analysis (Table 3), no significant differences were identified among the PABs on the basis of their aroma/flavor profile.

## 4. Conclusions

Fermentations at 25 °C of pomegranate juice initially fortified with added sugars from different sources generally resulted in products with higher TFC and FRSA values, most likely due to the lower fermentation period and therefore a shorter exposure of phenolic compounds to degradative processes. Instead, the compositional data for the fermented products at 15 °C revealed higher TPC and TMAC, implying that flavonoids may be more susceptible to degradation at 15 °C. As far as the type of sugar used for pomegranate juice fortification, the addition of CPJ is of great interest, as it exhibited an enhanced compositional profile for the fermented products (highest values for TPC, FRSA, and TMAC). The frequently employed approach for substrate fortification using pure S as an additional carbon source does not seem to offer any advantage other than to increase the alcohol content in the fermented product. In contrast, the CPJ, as a fermentable sugar source, not only compositionally fits with the pomegranate juice matrix but greatly enhances the potential health benefits of both raw material and the end-product, in view of its well-known bioactive components. The selection of an appropriate fermentation temperature was not clearly identified since no effects on aroma compounds were noted among various treatments. Although the results of the present study are important for the design and development of novel fermentation products with enhanced compositional-functional characteristics, more research is needed, especially for optimization on the organoleptic characteristics to improve consumer acceptability and marketability of this new line of products. In this context, the increased acidity of CPJ, when employed as an additional carbon source, remains of great concern.

## Figures and Tables

**Table 1:** Effect of sugar type addition and fermentation temperature on the physicochemical characteristics of PABs.

PAB	Reducing Sugars (g D-glucose L^−1^)	Ethanol (% *v*/*v*)	Glycerol (g L^−1^)	pH	Volatile Acidity (g Acetic Acid L^−1^)	Titratable Acidity (g Citric Acid L^−1^)
UPJ	112.8 ± 1.2			3.12 ± 0.01	0.1 ± 0.1	16.0 ± 0.1
Fermentation temperature 15 °C						
S	5.0 ± 0.1 ^b^	9.6 ± 1.5	5.4 ± 0.9	3.09 ± 0.01 ^ab^	0.9 ± 0.1 ^ab^	16.8 ± 0.3 ^a^
CPJ	5.7 ± 0.1 ^c^	9.3 ± 1.1	5.6 ± 0.7	3.03 ± 0.02 ^a^	0.8 ± 0.1 ^a^	23.2 ± 0.4 ^b^
CGJ	8.4 ± 0.1 ^e^	7.9 ± 0.4	4.8 ± 0.1	3.17 ± 0.02 ^bc^	0.9 ± 0.1 ^ab^	17.2 ± 0.1 ^a^
H	7.4 ± 0.3 ^d^	10.0 ± 0.4	5.8 ± 0.1	3.06 ± 0.01 ^a^	1.2 ± 0.1 ^b^	16.8 ± 0.2 ^a^
Fermentation temperature 25 °C						
S	4.1 ± 0.1 ^a^	9.8 ± 0.2	5.6 ± 0.3	3.18 ± 0.01 ^c^	0.9 ± 0.1 ^ab^	16.7 ± 0.5 ^a^
CPJ	4.9 ± 0.1 ^b^	9.4 ± 0.1	6.1 ± 0.1	3.17 ± 0.02 ^bc^	0.8 ± 0.1 ^a^	23.8 ± 0.2 ^b^
CGJ	7.5 ± 0.1 ^d^	8.9 ± 0.5	6.0 ± 0.4	3.29 ± 0.04 ^d^	0.9 ± 0.1 ^ab^	17.6 ± 0.2 ^a^
H	7.3 ± 0.1 ^d^	9.8 ± 0.4	5.7 ± 0.1	3.10 ± 0.01 ^abc^	0.9 ± 0.1 ^ab^	17.5 ± 0.3 ^a^
Significance of effect						
temperature	***	ns	ns	***	*	*
type of sugar	***	ns	ns	***	ns	ns

^a–e^ Different letters in the same column (UPJ excluded) indicate significant differences between means (*p* < 0.05). UPJ, unfermented pomegranate juice; S, PAB with added sucrose; CPJ, PAB with added concentrated pomegranate juice; CGJ, PAB with added concentrated grape juice; H, PAB with added honey; PAB, pomegranate alcoholic beverage; ns, not significant * *p* < 0.05; *** *p* < 0.001.

**Table 2:** Effect of sugar type addition and fermentation temperature on the antioxidant activity and phenolic compounds of PABs.

PAB	TFC (mg QE L^−1^)	TPC (mg GAE L^−1^)	FRSA (mM TRE)	TMAC (mg Cy3GE L^−1^)
UPJ	530.1 ± 7.3 ^d^	2478.3 ± 20.5 ^e^	27.1 ± 0.1 ^e^	149.0 ± 0.1 ^i^
Fermentation temperature 15 °C				
S	272.6 ± 20.4 ^a^	1318.8 ± 44.3 ^b^	13.8 ± 0.1 ^a^	106.0 ± 0.1 ^f^
CPJ	537.9 ± 11.7 ^d^	2302.8 ± 14.8 ^d^	19.3 ± 0.1 ^d^	144.0 ± 0.1 ^h^
CGJ	765.3 ± 8.8 ^f^	1801.0 ± 83.6 ^c^	14.2 ± 0.2 ^ab^	93.4 ± 0.1 ^b^
H	364.4 ± 4.4 ^bc^	1338.5 ± 44.3 ^b^	14.1 ± 0.1 ^ab^	102.5 ± 0.1 ^d^
Fermentation temperature 25 °C				
S	332.4 ± 24.8 ^b^	915.4 ± 64.0 ^a^	14.2 ± 0.2 ^ab^	104.5 ± 0.1 ^e^
CPJ	680.8 ± 5.8 ^e^	1668.2 ± 9.8 ^c^	19.8 ± 0.2 ^d^	131.5 ± 0.1 ^g^
CGJ	886.3 ± 21.9 ^g^	1407.4 ± 64.0 ^b^	15.1 ± 0.4 ^c^	85.7 ± 0.1 ^a^
H	392.1 ± 14.6 ^c^	866.2 ± 113.2 ^a^	14.6 ± 0.2 ^bc^	96.2 ± 0.1 ^c^
Significance of effect				
temperature	***	***	***	***
type of sugar	***	***	***	***

^a–i^ Different letters in the same column indicate significant differences between means (*p* < 0.05). UPJ, unfermented pomegranate juice; S, PAB with added sucrose; CPJ, PAB with added concentrated pomegranate juice; CGJ, PAB with added concentrated grape juice; H, PAB with added honey; PAB, pomegranate alcoholic beverage; TFC, total flavonoids content; TPC, total phenolics content; FRSA, free radical-scavenging activity, TMAC, total monomeric anthocyanin content; QE, quercetin equivalents; GAE, gallic acid equivalents; TRE: Trolox equivalents; Cy3GE, cyanidin-3-glucoside equivalents; *** *p* < 0.001.

**Table 3:** Effect of sugar type addition and fermentation temperature on aroma-related compounds (mg L^−1^) of PABs.

Compounds	PABs With Added Sugar								Significance	
	S		CPJ		CGJ		H		Temp	Sugar Type
	15 °C	25 °C	15 °C	25 °C	15 °C	25 °C	15 °C	25 °C		
**Esters**										
ethyl acetate	5.19 ± 0.83	4.09 ± 0.02	6.40 ± 0.56	3.75 ± 0.64	3.55 ± 3.04	3.09 ± 0.13	4.95 ± 2.05	3.81 ± 1.85		
ethyl propanoate	0.05 ± 0.07	Nd	0.15 ± 0.07	0.10 ± 0.00	0.05 ± 0.07	Nd	0.10 ± 0.00	Nd	*	*
ethyl butyrate	0.35 ± 0.21	0.20 ± 0.14	0.50 ± 0.14	0.30 ± 0.00	0.25 ± 0.07	Nd	0.36 ± 0.06	Nd	**	*
3-methylbutyl acetate	1.52 ± 0.26	2.76 ± 0.79	2.40 ± 1.55	2.90 ± 0.00	0.81 ± 0.13	1.23 ± 0.24	1.65 ± 0.35	1.45 ± 0.21		*
2-methylbutyl acetate	0.06 ± 0.08	0.14 ± 0.20	0.17 ± 0.10	0.10 ± 0.14	0.04 ± 0.04	Nd	0.07 ± 0.10	Nd		
ethyl hexanoate	2.46 ± 1.33	2.65 ± 0.35	2.49 ± 1.45	2.00 ± 0.28	1.10 ± 0.00	0.99 ± 0.15	2.63 ± 0.04	1.45 ± 0.78		
ethyl octanoate	4.44 ± 4.63	2.30 ± 0.29	3.53 ± 0.23	1.87 ± 1.24	1.11 ± 0.60	1.22 ± 0.59	2.49 ± 0.26	2.23 ± 1.71		
ethyl phenylacetate	Nd	Nd	Nd	0.15 ± 0.07	Nd	Nd	Nd	Nd		
2-phenylethyl acetate	0.76 ± 0.91	0.95 ± 0.21	0.50 ± 0.42	1.05 ± 0.64	0.35 ± 0.21	1.05 ± 0.19	0.40 ± 0.00	1.35 ± 0.49	*	
ethyl decanoate	1.10 ± 1.13	0.10 ± 0.14	1.20 ± 0.57	0.70 ± 0.71	0.30 ± 0.28	0.10 ± 0.14	0.25 ± 0.07	0.45 ± 0.64		
ethyl dodecanoate	1.10 ± 1.27	Nd	0.80 ± 0.42	0.35 ± 0.35	Nd	Nd	Nd	Nd		
ethyl tetradecanoate	Nd	Nd	0.15 ± 0.07	Nd	Nd	Nd	Nd	Nd		
ethyl hexadecanoate	4.00 ± 4.81	0.55 ± 0.35	1.65 ± 1.48	1.95 ± 2.05	0.25 ± 0.21	0.35 ± 0.21	0.45 ± 0.07	2.30 ± 1.27		
Total esters	21.02 ± 3.34	13.74 ± 0.75	19.95 ± 0.01	15.22 ± 5.84	7.80 ± 1.97	8.03 ± 1.96	13.34 ± 2.59	13.04 ± 3.26		**
**Fatty acids**										
octanoic acid	1.70 ± 1.53	0.57 ± 0.38	1.94 ± 0.21	3.76 ± 2.51	2.31 ± 0.52	2.08 ± 0.96	2.57 ± 2.17	0.95 ± 0.07		
decanoic acid	Nd	Nd	Nd	1.30 ± 1.27	0.10 ± 0.14	Nd	0.30 ± 0.28	Nd		
Total fatty acids	1.70 ± 1.53	0.57 ± 0.38	1.94 ± 0.21	5.06 ± 3.78	2.41 ± 0.38	2.08 ± 0.96	2.87 ± 2.45	0.95 ± 0.07		
**Alcohols**										
2-methyl-1-propanol	0.25 ± 0.21	0.51 ± 0.15	1.01 ± 0.98	0.25 ± 0.21	0.38 ± 0.32	0.53 ± 0.04	0.50 ± 0.14	0.30 ± 0.28		
3-methyl-1-butanol	18.00 ± 1.94	17.51 ± 1.68	24.22 ± 5.06	16.71 ± 4.38	18.22 ± 15.10	17.55 ± 1.10	19.69 ± 5.71	14.52 ± 2.83		
2-methyl-1-butanol	5.96 ± 1.20	6.21 ± 0.15	5.10 ± 0.01	4.57 ± 1.04	5.96 ± 4.72	6.65 ± 1.27	7.14 ± 1.48	5.09 ± 0.83		
2,3-butanediol	0.45 ± 0.64	Nd	0.75 ± 1.06	0.30 ± 0.42	0.90 ± 1.27	1.08 ± 0.96	0.42 ± 0.59	Nd		
(Z)-3-hexen-1-ol	0.05 ± 0.07	Nd	Nd	0.05 ± 0.06	Nd	Nd	Nd	Nd		
1-hexanol	0.05 ± 0.07	Nd	Nd	0.05 ± 0.06	Nd	Nd	Nd	Nd		
2-phenylethanol (phenylethyl alcohol)	4.80 ± 4.95	2.40 ± 0.42	2.00 ± 0.01	5.30 ± 3.25	3.55 ± 2.90	8.50 ± 3.11	2.75 ± 1.06	9.85 ± 6.58		
Total alcohols	29.56 ± 3.22	26.62 ± 1.81	33.08 ± 7.12	27.21 ± 1.82	29.01 ± 9.07	34.31 ± 6.48	30.49 ± 2.72	29.76 ± 2.63		

Nd, not detected; S, PAB with added sucrose; CPJ, PAB with added concentrated pomegranate juice; CGJ, PAB with added concentrated grape juice; H, PAB with added honey; PAB, pomegranate alcoholic beverage, * *p* < 0.05; ** *p* < 0.01.

## Data Availability

All data are contained within the article.

## Supplementary Materials

The following are available online at https://www.mdpi.com/article/10.3390/antiox10060889/s1, Table S1: Aroma-related compounds (mg L^−1^) of pomegranate-based juices fortified with different sugar containing materials as additional fermentable carbohydrate sources, Figure S1: Principal component analysis (PCA) plot of minor volatiles detected in juices and PABs fortified with the addition of different types of sugars to enhance fermentability of the raw material, at 15 °C and 25 °C.
