# Supplementary material for: Impact of Sugar Type Addition and Fermentation Temperature on Pomegranate Alcoholic Beverage Production and Characteristics
Source: Antioxidants (Basel). 2021 Jun 1;10(6):889. doi: 10.3390/antiox10060889 (PMC8228759; doi:10.3390/antiox10060889)
Supplement: Supplementary file 1 [file antioxidants-10-00889-s001.zip › antioxidants-1229612-supplementary.pdf]

Table S1. Aroma-related compounds (mg L^{-1}) of pomegranate-based juices fortified with different sugar containing materials as additional fermentable carbohydrate sources.

Compounds	PJ	PJ+S	PJ+CPJ	PJ+CGJ	PJ+H
**Esters**					
ethyl acetate	0.1	0.1	0.1	0.1	0.1
ethyl octanoate	Nd	Nd	Nd	Nd	0.1
**Alcohols**					
2-methyl-1-propanol	<0.1	<0.1	<0.1	<0.1	<0.1
3-methyl-1-butanol	0.2	0.1	0.1	0.1	0.1
2-methyl-1-butanol	0.1	0.1	<0.1	<0.1	<0.1
3-methyl-2-buten-1-ol	Nd	<0.1	Nd	<0.1	Nd
(z)-3-hexen-1-ol	0.5	<0.1	0.5	0.2	0.5
1-hexanol	0.5	0.4	0.5	0.3	0.5
2-phenylethanol (phenylethyl alcohol)	<0.1	<0.1	<0.1	<0.1	<0.1
a-terpineol	0.1	<0.1	<0.1	<0.1	0.1
2,4-bis(1,1-dimethylethyl)-phenol	<0.1	<0.1	<0.1	<0.1	<0.1

Nd, Not detected; PJ, pomegranate juice; PJ+S, pomegranate juice with added sucrose (20° Brix, final content); PJ+CPJ, pomegranate juice with added concentrated pomegranate juice (20° Brix, final content); PJ+CGJ, pomegranate juice with added concentrated grape juice (20° Brix, final content); PJ+H, pomegranate juice with added honey (20° Brix, final content).

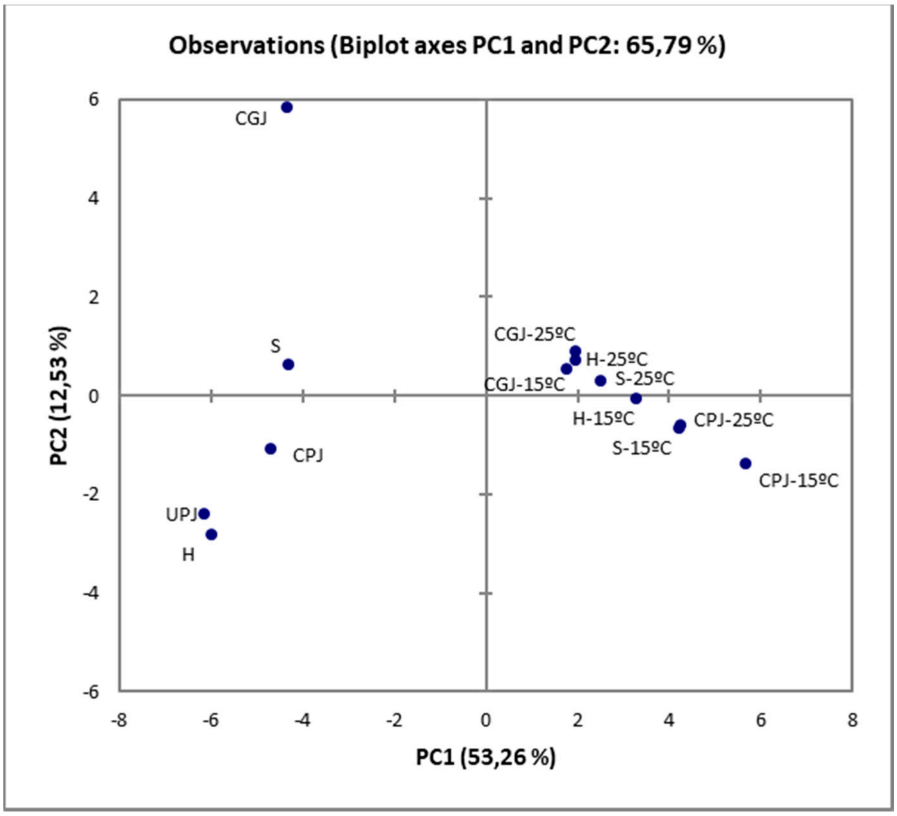

Figure S1. Principal component analysis (PCA) plot of minor volatiles detected in juices and PABs fortified with the addition of different types of sugars to enhance fermentability of the raw material, at 15 °C and 25 °C. PABs, pomegranate alcoholic beverages; S, pomegranate juice with added sucrose; S-15ºC and S-25ºC, PABs with added sucrose fermented at 15 and 25ºC, respectively; CPJ, pomegranate juice with added concentrated pomegranate juice; CPJ-15ºC and CPJ-25ºC, PABs with added concentrated pomegranate juice fermented at 15 and 25ºC, respectively; CGJ, pomegranate juice with added concentrated grape juice; CGJ-15ºC and CGJ-25ºC, PABs with added concentrated grape juice fermented at 15 and 25ºC, respectively; H, pomegranate juice with added honey; H-15ºC and H-25ºC, PABs with added honey fermented at 15 and 25ºC, respectively.
